# A Metabolic Pattern in Healthy Subjects Given a Single Dose of Metformin: A Metabolomics Approach

**DOI:** 10.3389/fphar.2021.705932

**Published:** 2021-07-15

**Authors:** Lina A. Dahabiyeh, Muhammad Mujammami, Tawfiq Arafat, Hicham Benabdelkamel, Assim A. Alfadda, Anas M. Abdel Rahman

**Affiliations:** ^1^ Department of Pharmaceutical Sciences, School of Pharmacy, The University of Jordan, Amman, Jordan; ^2^ Endocrinology and Diabetes Unit, Department of Medicine, College of Medicine, King Saud University, Riyadh, Saudi Arabia; ^3^ University Diabetes Center, King Saud University Medical City, King Saud University, Riyadh, Saudi Arabia; ^4^ Jordan Center for Pharmaceutical Research, Amman, Jordan; ^5^ Proteomics Resource Unit, Obesity Research Center, College of Medicine, King Saud University, Riyadh, Saudi Arabia; ^6^ Metabolomics Section, Department of Clinical Genomics, Center for Genome Medicine, King Faisal Specialist Hospital and Research Centre (KFSHRC), Riyadh, Saudi Arabia; ^7^ Department of Biochemistry and Molecular Medicine, College of Medicine, Al Faisal University, Riyadh, Saudi Arabia; ^8^ Department of Chemistry, Memorial University of Newfoundland, St. John’s, NL, Canada

**Keywords:** metabolomics, metformin, branched-chain amino acids, mass spectrometry, glycerophospholipid, eicosanoids, type 2 diabetes mellitus, cancer

## Abstract

Metformin is a widely prescribed medication for the treatment of type 2 diabetes mellitus (T2DM). It possesses effective roles in various disorders, including cancer, dyslipidemia, and obesity. However, the underlying mechanisms of metformin's multiple benefits are not fully understood. Herein, a mass spectrometry-based untargeted metabolomics approach was used to investigate the metabolic changes associated with the administration of a single dose of metformin in the plasma of 26 healthy subjects at five-time points; pre-dose, before the maximum concentration of metformin (C_max_), C_max_, after C_max_, and 36 h post-dose. A total of 111 metabolites involved in various biochemical processes were perturbed, with branched-chain amino acid (BCAA) being the most significantly altered pathway. Additionally, the Pearson similarity test revealed that 63 metabolites showed a change in their levels dependent on metformin level. Out of these 63, the level of 36 metabolites was significantly altered by metformin. Significantly altered metformin-dependent metabolites, including hydroxymethyl uracil, propionic acid, glycerophospholipids, and eicosanoids, pointed to fundamental biochemical processes such as lipid network signaling, energy homeostasis, DNA lesion repair mechanisms, and gut microbiota functions that could be linked to the multiple beneficial roles of metformin. Thus, the distinctive metabolic pattern linked to metformin administration can be used as a metabolic signature to predict the potential effect and mechanism of actions of new chemical entities during drug development.

## Introduction

Diabetes is a severe chronic disease affecting hundreds of millions of people worldwide, leading to high mortality and morbidity rates and increased health care costs (Guasch-Ferre et al., 2016). Metformin, dimethyl biguanide, is one of the most prescribed drugs for treating type 2 diabetes mellitus (T2DM) worldwide (Davies et al., 2018; Buse et al., 2020). It is an effective, safe, and relatively inexpensive anti-hyperglycemic agent associated with improved glycemic control and insulin sensitivity (Song, 2016). However, metformin does not alter glucose homeostasis in non-diabetic subjects (Sanchez-Rangel and Inzucchi, 2017). In addition, it has demonstrated cardioprotective effects that were not associated with clinical hypoglycemia or/and body weight gains (Sanchez-Rangel and Inzucchi, 2017; Foretz et al., 2019).

Metformin exerts its antidiabetic action mainly by acute suppression of hepatic gluconeogenesis through a transient inhibition of the mitochondrial respiratory chain complex I with a consequence activation of adenosine monophosphate (AMP)-activated protein kinase (AMPK) through liver kinase B1 (Viollet et al., 2012; Rena et al., 2017). Besides, metformin acts on multiple tissues and targets different pathways. It has been reported to reduce oxidative stress mainly by inhibiting the production (or neutralizing) of mitochondrial reactive oxygen species (ROS) (Bonnefont-Rousselot et al., 2003; Kane et al., 2010). Several disordered and complications are controlled and improved by metformin, including; metabolic and reproductive abnormalities of polycystic ovary syndrome (PCOS), cardiovascular complications associated with diabetes, cancer prognosis, and neurodegenerative diseases (Viollet et al., 2012; Rotermund et al., 2018; Foretz et al., 2019). Additionally, clinical studies have shown that metformin has beneficial effects on systemic inflammatory markers (Cameron et al., 2016) and weight loss in insulin-sensitive and insulin-resistant overweight and obese patients (Seifarth et al., 2013).

The pleiotropic properties of metformin and its numerous therapeutic areas suggest that various underlying mechanisms and metabolic pathways could be involved. Despite being introduced into the market for over 60 years, the mechanism of action of metformin remains partially explored and understood (Viollet et al., 2012; Foretz et al., 2014; Foretz et al., 2019). This urges the need for new and considerable efforts to understand better the cellular and molecular mechanisms of action of metformin.

Metabolomics is the comprehensive analysis of a set of small molecules (i.e., amino acids, lipids, and carbohydrates), referred to as metabolites within cells, biofluids, tissues, or organisms. It is a powerful analytical tool that is widely used to provide rich mechanistic information on drugs, and aid in identifying potential biomarkers that can be used to monitor the efficacy of drug therapies (Balashova et al., 2018; Jacob et al., 2019; Dahabiyeh et al., 2021). Pharmacometabolomics is an effective approach to capture the metabolic signatures linked to drug exposure and, therefore, improves the understanding of their underlying mechanisms of actions and allows individual differences recognition and drug toxicity prediction (Adam et al., 2016; Malkawi et al., 2018; Dahabiyeh et al., 2020). Several studies have reported the use of metabolomics to investigate the effect of metformin under pathological conditions, including T2DM (Bao et al., 2009; Adam et al., 2016), obesity (Zhu et al., 2013), cancer (Liu et al., 2016), metabolic syndrome (Ryan et al., 2020), and PCOS (Vinaixa et al., 2011). In this study, a single dose of metformin was given to healthy subjects. A label-free mass spectrometry-based untargeted metabolomics approach was used to identify metabolic dysregulation and pathways associated with metformin excreated levels (metformin-dependent metabolites). The dysregulated metformin-dependent metabolites could provide novel insights into the underlying biological pathways impacted by metformin administration.

## Materials and Methods

### Subject Recruitment and Study Design

Subject recruitment and blood sample collection were conducted at Jordan Center for Pharmaceutical Research in Amman, Jordan. The Institutional Review Board reviewed and approved the study at Jordan Center for Pharmaceutical Research at Amman, Jordan (IRB-01-R02), and Institutional Review Board (IRB) at King Saud University (approval number E-19-4234) and written informed consent was obtained from all participants. Twenty-six healthy male subjects, aged 18–50 years, were enrolled in the study, whereas individuals with recorded health conditions were excluded. Each subject received a single oral dose of 500 mg metformin hydrochloride film-coated tablet under standard fed conditions. A night before drug administration, all subjects were served standard dinner at least 10 h before metformin intake. Standard breakfast was served 30 min before drug administration, while standard lunch and dinner were served 6 and 12 h post drug administration. Individual serum concentrations of metformin were assayed at different time points following drug administration to determine C_max_ (unpublished data). For each participant, blood samples were collected in heparinized tubes at multiple time points, and only five-time points were selected for this metabolomics study; pre-drug administration, 1.5 h before the maximum concentration of metformin in the serum (C_max_), C_max_, 2 h after C_max_, and 36 h post-drug administration, culminating a total of 130 samples. In this study, we selected the above five-time points to give a comprehensive measure of the metabolic pattern of metformin.

Screening (up to 14 days pre-dose administration) and follow-up (up to 7 days post-dose administration) examinations including comprehensive medical history, electrocardiogram (ECG), physical examination, vital signs (blood pressure and heart rate) measurements, blood hematology, and blood chemistry were performed for all participants. HbA1c, urinalysis, and serology tests (HBs Ag, HCV Ab, HIV I andII) were only performed during the screening examination. In addition, blood glucose levels were monitored at different time points. The demographic and clinical data of the participants are presented in Table 1.

**TABLE 1:** Clinical and demographic data of recruited subjects (*n* = 26 male) during screening and follow-up periods.

Clinical and demographic data	Mean ± SD	
	Screening	Follow-up
Body mass index (BMI, kg/m^2^), (range)	25 ± 3.8, (19.2–29.3)	—
Age (years)	31 ± 9.2	—
Blood pressure (mm Hg)	≤120/80	≤120/80
Heart rate (beat/minute)	69.6 ± 4	71.9 ± 6.5
Biochemistry		
Glucose (mg/dl)	98.2 ± 7.9	91.5 ± 9.7
Urea (mg/dl)	29.8 ± 5.9	31.3 ± 6.7
Creatinine (mg/dl)	1.04 ± 0.14	1.08 ± 0.11
Sodium (mmole/L)	143.2 ± 2.7	143 ± 1.9
Potassium (mmole/L)	4.3 ± 0.2	4.2 ± 0.17
GOT (u/L)	21.2 ± 6.9	26 ± 17.0
GPT (u/L)	26.2 ± 11.1	30 ± 34.0
ALK^a^ (u/L)	105 ± 19.0	86 ± 16.0
Total protein (g/dl)	7.4 ± 0.5	7.7 ± 0.5
Total bilirubin (mg/dl)	0.47 ± 0.14	0.5 ± 0.5
HbA1c (%)	5.2 ± 0.23	—
Hematology		
Hemoglobin (g/dl)	16.1 ± 1.0	15.8 ± 1.1
Hematocrit (%)	47.7 ± 2.9	45.6 ± 2.8
R.B.C (1012/L)	5.4 ± 0.4	5.3 ± 0.3
M.C.V (fL)	88.2 ± 4.3	86.6 ± 3.5
M.C.H (pg)	29.6 ± 1.8	30.0 ± 1.6
M.CH.C (g/dl)	33.6 ± 0.6	34.6 ± 0.7
Differential Leucocytes Count		
Leucocytes (10^9^/L)	7.8 ± 2.0	
Neutrophils (%)	62.3 ± 3.7	64.2 ± 2.5
Lymphocytes (%)	33.5 ± 3.2	32.0 ± 2.5
Monocytes (%)	3.4 ± 1.1	3.0 ± 0.7
Eosinophils (%)	0.73 ± 0.7	0.77 ± 0.8
Basophils (%)	0.04 ± 0.2	0.12 ± 0.3
Platelets (10^9^/L)	245.1 ± 40.5	272.1 ± 46.2

Data are presented as mean ± standard deviation.

GOT: glutamic oxaloacetic transaminase, GPT: glutamic pyruvic transaminase, ALK: Alkaline phosphatase, R.B.C: red blood cell, M.C.V mean corpuscular volume, M.C.H mean corpuscular hemoglobin, M.CH.C: mean corpuscular hemoglobin concentration.

All lab tests were within the normal range with no significant difference between screening and follow-up periods with the exception of ALK.

a Lab test values were significantly different between screening and follow-up periods (independent *t*-test, *p* value ≤ 0.05).

### Sample Preparation and Metabolite Extraction

A total of 130 serum samples were subjected to label-free untargeted metabolomics analysis using high-resolution liquid chromatography-mass spectrometry (LC-MS). Metabolites were extracted as previously described (Aleidi et al., 2021). Briefly, to 100 μL serum sample, 300 μL of methanol and 10 μL of 2.8 mg/ml DL-o-chlorophenylalanine internal standard were added. After centrifugation for 15 min, the supernatant was mixed with cold acetonitrile (ACN) and centrifuged again for 5 min. Finally, the supernatant was dried, and before LC-MS analysis, the dried residue was reconstituted in 1:1 (v/v) methanol/water.

### Label-free Liquid Chromatography-Mass Spectrometry Untargeted Metabolomics Analysis

Metabolomics profiling was performed by Ultimate 3000 LC combined with Q Exactive MS (Thermo Fisher Scientific, CA, United States) as reported in our recent publication (Aleidi et al., 2021). Briefly, extracted metabolites were first separated using an ACQUITY UPLC HSS T3 (100 × 2.1 mm 1.8 μm) and a mobile phase composed of solvent A (0.05% formic acid-water) and solvent B (ACN) with a gradient elution over 16 min applied at 300 μl/min flow rate. MS spectra were acquired in full MS scan in the range *m/z* 50–1,500, with 25,000 enhanced mass resolution and a frequency 15 spectra per second. The capillary voltage was 3000 and 3200 V for positive and negative ionization modes, respectively. The fragmentation was achieved for MSMS experiments at 175 V, with nebulizer gas at 35 bsi, and gas temperature 450°C. Chromatographic and MS parameters (under positive and negative ionization modes) were kept as reported previously (Aleidi et al., 2021). Pooled samples prepared the quality control (QC) sample. A QC injection was performed every 10 LC-MS sample runs. In total, there were 18 QC samples injected and analyzed.

### Metabolites Identification

The raw data were acquired and aligned using the compound discoverer software (Thermo Fisher Scientific, United States) based on the *m/z* value and the ion signals’ retention time. Then, the chemical structures of metabolites were identified by matching the data obtained from accurate mass analysis and MS/MS fragmentation with data available in the online databases; the Human Metabolome Database (www.hmdb.ca), METLIN (www.metlin.scripps.edu), and the Mass Bank (www.massbank.jp).

### Statistical Analysis

Multivariate statistical analysis was performed using SIMCAP+14 from Umetrics AB (Umeå, Sweden). For the identified metabolites, peak intensities at each time point were normalized, Parito-scaled, and log-transformed. The processed peak intensities were used to generate principal component analysis (PCA) and orthogonal partial least squares-discriminant analysis (OPLS-DA) models to overviewing the metabolic differences between the various time points. The robustness of the created models was evaluated by the fitness of model (R^2^Y) and predictive ability (Q^2^) values (Worley and Powers, 2013). MetaboAnalyst version 4.0 (McGill University, Montreal, Canada) (http://www.metaboanalyst.ca) was used to visualize the affected metabolic pathways as of metformin intake (Chong et al., 2018).

Univariate analysis using one-way analysis of variance (ANOVA) and post-hoc Tukey’s analysis method was performed for time points. Significantly differentially expressed metabolites were determined based on false discovery rate (FDR) adjusted *p*-value less than 0.05 and fold change (FC > 1.5, <0.67). The total sample median was used to normalize the signal, ensure normal distribution, and represent Z-score. The metabolic patterns connected to metformin's action were developed using Venn diagrams and Pearson similarity test (MPP Software, Agilent Inc., CA) as discussed before (Gu et al., 2020).

## Results

### Clinical and Demographic Data of Study Subjects

Table 1 summarizes the demographic and clinical data of the 26 male subjects included in this study. All participants were healthy non-diabetic men. Screening and follow-up examinations revealed that all subjects had normal heart rates, blood pressure (≤120/80 mm Hg), blood glucose, and HbA1c levels. Hematology and biochemistry tests were within the normal range for all participants during screening and follow-up examinations with no significant difference between the two periods except for the alkaline phosphatase test (ALK known as ALP), Table 1. The level of the ALK enzyme was significantly lower in the follow-up period (105 ± 19) compared to the screening period (86 ± 16), which indicates that metformin can affect the circulating levels of ALK. Of note, one subject showed a substantial increase in the levels of the two liver enzymes, glutamic oxaloacetic transaminase (GOT, known as AST) and glutamic pyruvic transaminase (GPT, known as ALT), during the follow-up period compared to screening tests (15, 19 in screening to 104, 187 in follow up, respectively). Moreover, the same subject was the only one to show an increase in ALK level compared to others who showed decreased ALK levels during the follow-up period.

### Metabolites Detection and Multivariate Analysis

**FIGURE 1:**
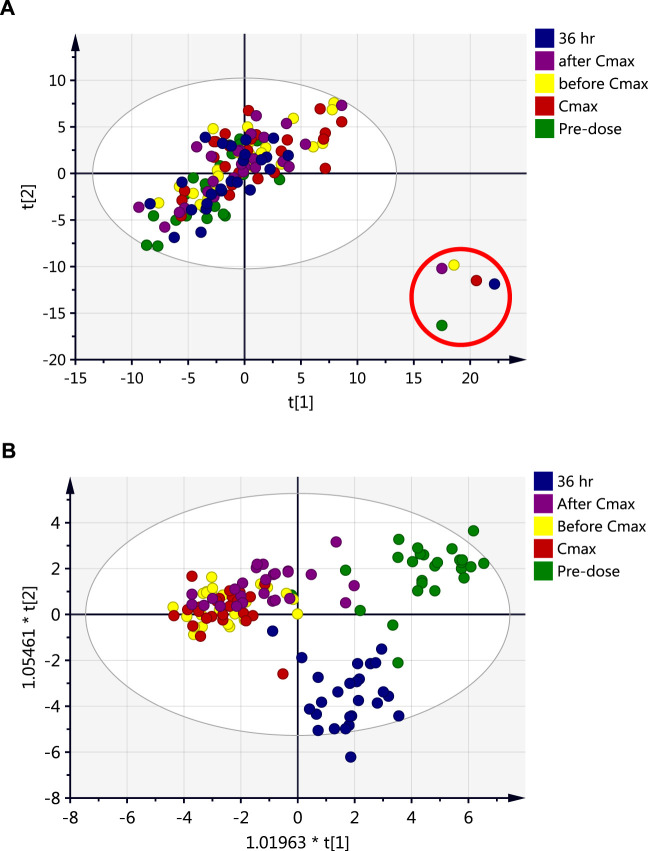
Scores plots of the metabolite profile of serum samples obtained from 26 healthy subjects after single dose of metformin at five time points; pre-dose (baseline level, green), and 1.5 h before C_max_ (yellow), C_max_ (red), 2 h after C_max_ (purple) and 36 h post-drug administration (blue). **(A)** PCA (R_2_X = 0.71, Q^2^ = 0.44), **(B)** OPLS-DA (R2X = 0.28, R2Y = 0.40, Q2 = 0.27).

A total of 4,456 and 3,182 mass ion features were detected in positive and negative ionization modes, respectively. Using the data of accurate masses and MS/MS fragments, 444 and 400 metabolites were putatively identified, in positive and negative ionization modes, respectively, with 110 metabolites commonly identified in both ionization modes. Metabolites identified by the two ionization modes were merged (734 metabolites) and exported for multivariate analysis using PCA and OPLS-DA (Figure 1). The data were deposited in MetaboLight (accession Number MTBLS2949.

Individual serum concentrations of metformin were assayed at different time points following drug administration to determine C_max_ (unpublished data). Five-time points reflecting different metformin levels in the circulation were selected; pre-dose and four post-drug administration time points (before C_max_, C_max_, after C_max_, and 36 h) for endogenous metabolomics association. Multivariate analysis (Figure 1) was performed to investigate group clustering and separation and identify potential outliers among the compared data time points. The PCA score plot (Figure 1A) overlaps with no separation or clustering achieved between the various time points. However, the PCA model clearly identified outliers that belong to the metabolic data of one subject at the five-time points (Figure 1A). The same subject had an increased level of liver enzymes during the follow-up period. The different metabolic patterns of this subject might be independent of metformin administration as all time-points, including pre-dose, are clustered outside the PCA model’s confidence interval. Therefore, data for this participant were excluded from the dataset before further analyses.

The OPLS-DA model could not entirely separate the dataset obtained at different time points, and only partial separation could be noticed mainly due to the complexity of the dataset (Figure 1B). demonstrates that pre-dose and 36 h post-dose samples exhibited evident separation from the other time points. In contrast, the remaining three-time points (before C_max_, C_max,_ and after C_max_) were overlapped and did not show any separation or clustering. The previous findings indicate that the metabolic profile after 36 h of metformin administration did not retain a level close to the baseline. However, the circulating level of metformin could not be detected at 36 h post-administration (data not shown). Therefore, it is plausible that a longer time is needed after the intake of metformin for the metabolite level to return to a normal level even if the level of metformin in the circulation is very low.

### The Effect of Metformin on Circulating Metabolites in Healthy Subjects

**FIGURE 2:**
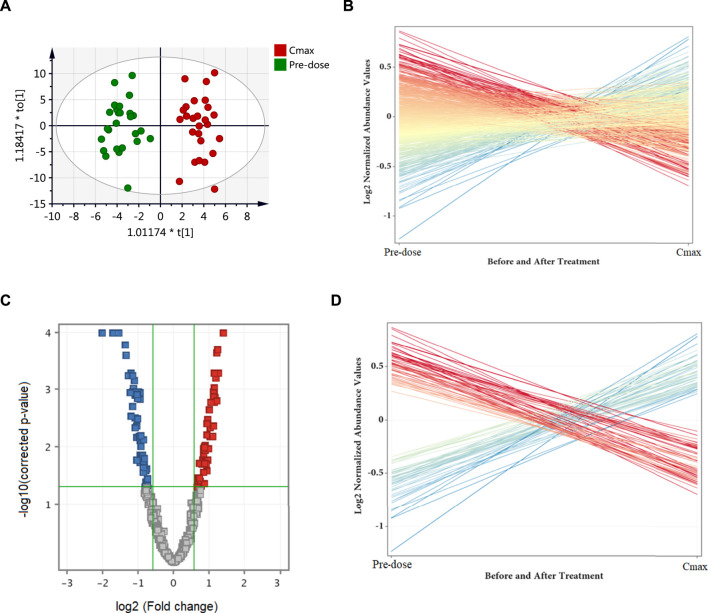
Differentially expressed metabolites as of metformin administration based on a binary comparison between baseline level (pre-dose) and C_max_ level. **(A)** OPLS-DA (R2X = 0.26, R2Y = 0.92, Q2 = 0.62) scores plot of the metabolic profile of serum samples obtained from 26 subjects pre-dose (green) and at C_max_ (red). **(B)** Out of the 734 identified metabolites, 111 were significantly dysregulated (presented as red and blue lines) while the remaining were unchanged (yellow lines). **(C)** Volcano plot of up (red, n = 57) and down (blue, n = 54) regulated metabolites. **(D)** The differentially altered metabolites as of metformin administration. Blue and red refer to up-and down-regulated metabolites, respectively.

**FIGURE 3:**
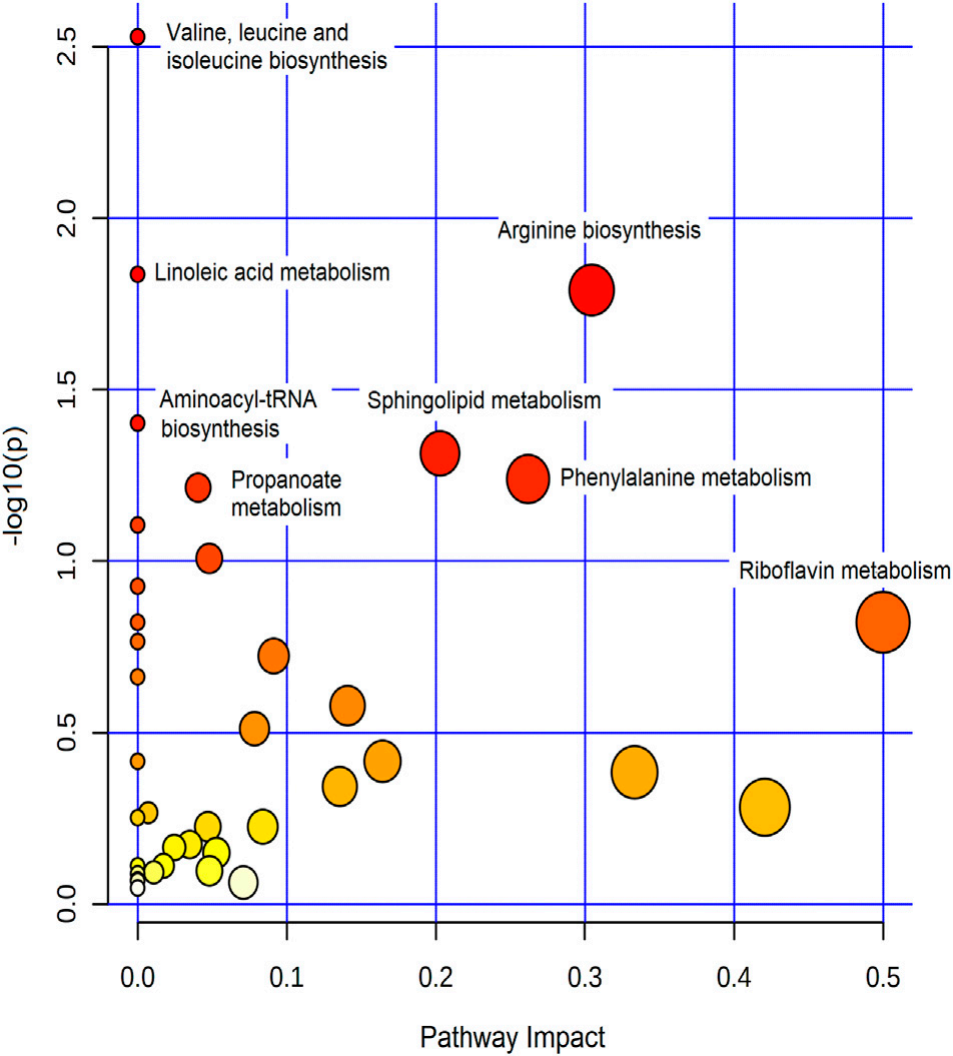
Summary of the most affected pathways. The node color and size are based on the *p*-value and the pathway impact value, respectively.

The effect of metformin on biological metabolites was examined by investigating its influence on the levels of the 734 putatively identified metabolites using mainly two-time points; pre-dose level (before treatment) and C_max_ (post-drug administration associated with the highest level of metformin), as presented in Figure 2. OPLS-DA model showed evident separation between the two groups (Figure 2A), indicating that metformin administration has significantly altered the dynamic of different metabolic processes. Binary comparison using volcano plot (cutoff 1.5) revealed that out of 734 metabolites, 111 were dysregulated, of which 57 and 54 metabolites were up-and down-regulated as of metformin administration, respectively (Figures 2C,D). The identity of these metabolites is presented in Supplemenatry Table S1 in the supplementary material. The 111 altered metabolites are involved in various biological processes, including valine, leucine, and isoleucine biosynthesis, linoleic metabolism, arginine biosynthesis, and aminolylacyl-tRNA biosynthesis, as shown in Figure 3.

### Endogenous Metabolites Linked to Metformin Level

**FIGURE 4:**
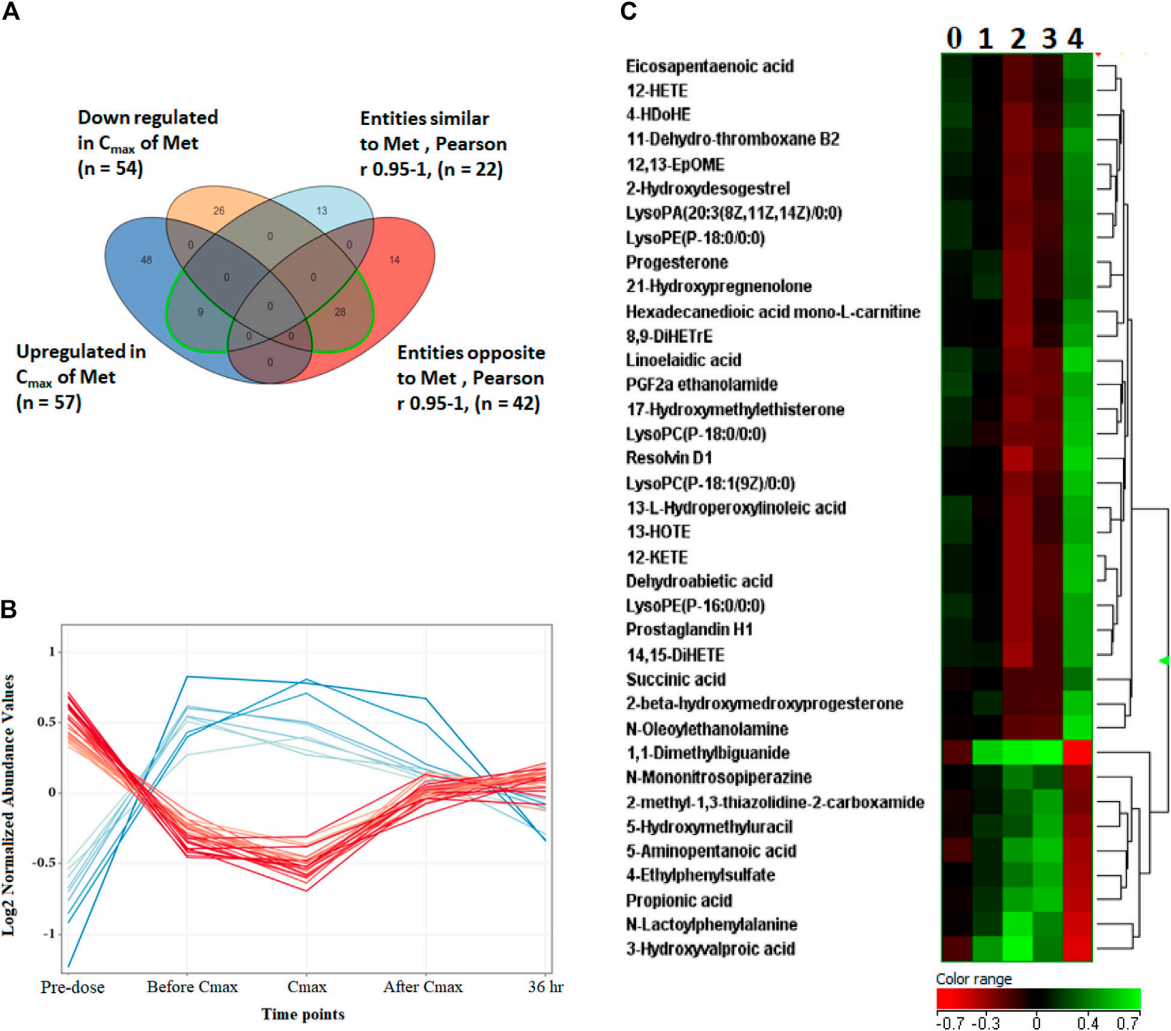
Metformin-dependent metabolites. **(A)** Venn diagram showing significantly altered metabolites and metabolites with similar or opposite change in their metabolic levels compared to metformin levels at the five time points based on Pearson similarity test (R0.95–1). **(B)** The 28 (red line) and 8 (blue line) metformin- dependent metabolites showing opposite and similar trend in their levels to metformin, respectively. **(C)** Hierarchal clustering (HAC) and heatmap analysis of significantly altered metformin dependent metabolites. Time points 0, 1, 2, 3, and 4 refer to baseline, before C_max_, C_max_, after C_max_ and 36 h post metformin administration. Metformin is 1,1-Dimethylbiguanide.

Metabolites dysregulated endogenously, and their levels associated with metformin’s level (Metformin-dependent metabolites) were identified by comparing the levels of differentially altered metabolites to metformin patterns at the five-time points. The Pearson similarity test (R = 0.95–1) revealed that 21 metabolites (excluding metformin) showed a similar expression trend to metformin Figure 4A. Out of these 21 metabolites, 8 (excluding metformin) significantly increased their levels based on the binary comparison between baseline and C_max_ levels (Figure 4B). These include 5-aminopentanoic acid, propionic acid, hydroxymethyl uracil, and ethyl phenyl sulphate (Figure 4C). On the other hand, 42 metabolites acted in an opposed manner to metformin levels, with 28 metabolites showing a significant decrease in C_max_ level compared to baseline. Metabolites that opposed levels to metformin patterns mainly involved arachidonic and linoleic acid metabolisms and included glycerophospholipids(such as lysophosphatidic acid and lysophospholipid) and eicosanoids (prostaglandin H1, 11-dehydrothromboxane B2), Figure 4C. Heatmap of the 36 metformin-dependent significantly perturbed metabolites and their levels at the five-time points is shown in Figure 4C.

**FIGURE 5:**
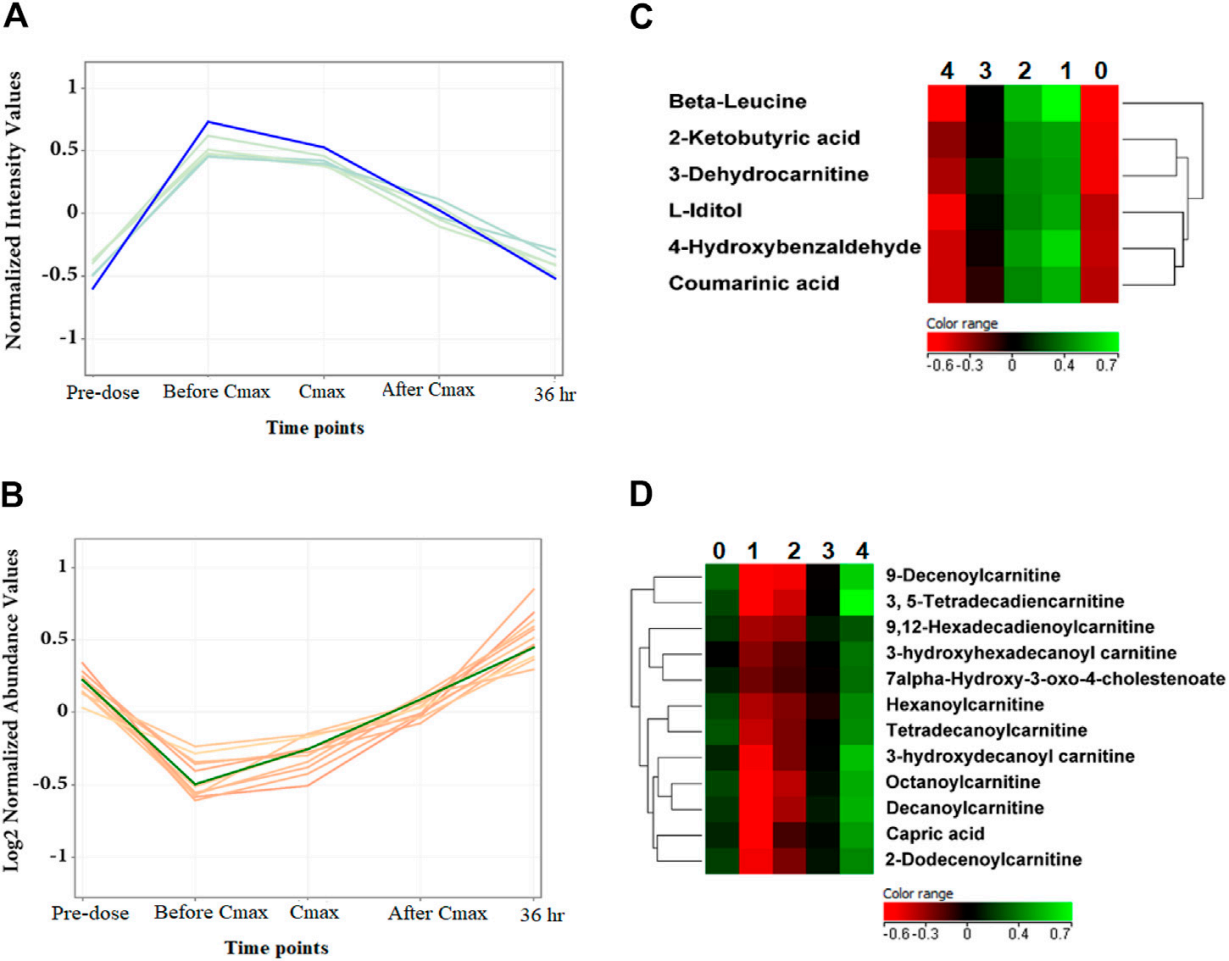
The level of metformin-independent metabolites at the five-time points. **(A, B)** The 6 and 12 metformin-independent metabolites, respectively, initially increased or decreased, followed by a subsequent linear decrease or increase in their metabolic levels. **(C, D)** Hierarchal clustering (HAC) and heatmap analysis of significantly altered metformin independent metabolites. Time points 0, 1, 2, 3, and 4 refer to baseline, before C_max_, C_max_, after C_max,_ and 36 h post metformin administration.

Noteworthy, specific metabolites showed an interesting change in their levels compared to the metformin pattern (Figure 5 **)**. After metformin administration, the level of 6 and 12 metabolites was initially increased and decreased, respectively, followed by a subsequent linear decrease or increase, until returning to levels similar to pre-dose at 36 h post drug administration as shown in Figures 5A,B, respectively. Among these metabolites are several acylcarnitines and coumaric acid.

## Discussion

Metformin is a widely used biguanide drug due to its outstanding safety profile, low cost, and promising effects in T2DM, cancer, PCOS, weight reduction, and many other medical conditions. It exerts multiple effects through different signaling pathways. Extensive literature has investigated the role of metformin in various disorders. However, the underlying mechanisms of its multiple benefits remain to be elucidated. Moreover, examining the effect of metformin under pathological conditions makes identifying metabolites specifically altered due to metformin rather than the disease state difficult, particularly that many patients will take metformin in combination with other medications, which will definitely affect the metabolomics data. To the best of our knowledge, this is the first study to examine the effect of a single dose of metformin on the metabolic pattern of healthy subjects at different time points.

Metformin induced significant changes in several biochemical pathways, including amino acids and aminoacyl-tRNA biosyntheses, and fatty acid metabolism. Amongst them, alteration of branched-chain amino acids, BCAA (valine, leucine, and isoleucine), was the most significant pathway (Figure 3). BCAA are crucial regulators of energy homeostasis, glucose and lipid metabolism, gut health, and immunity (Nie et al., 2018). In addition, they serve as substrates to synthesize nitrogenous compounds and play a critical role in protein and fatty acids syntheses (Ma et al., 2017; Nie et al., 2018). Therefore, metabolic imbalance in BCAA levels (increased catabolic flux or circulating levels) is associated with a range of conditions such as T2DM, obesity, cancer, and cardiovascular diseases (Nie et al., 2018; Siddik and Shin, 2019). The physiological roles of BCAA are mainly mediated via phosphoinositide 3-kinase/protein kinase B/mammalian target of rapamycin (PI3K/AKT/mTOR) signal pathway (Nie et al., 2018). Our findings indicate that one reason behind the multiple beneficial effects of metformin might be due to its significant impact on BCAA pathway. This is a plausible explanation since metformin has previously been reported to suppress BCAA catabolic enzyme expression or activity (Rivera et al., 2020) and PI3K/AKT/mTOR signal pathway (Zhao et al., 2018; Wang et al., 2019), which thereby will affect several crucial biochemical pathways.

Out of the 111 metabolites significantly altered by metformin administration, 36 metabolites displayed a change in their level similar or opposed to the metformin level pattern at the five-time points (Pearson similarity test (R = 0.95–1). Therefore, these metabolites were considered as metformin-dependent metabolites. Amongst the metabolites that had a similar pattern to metformin is 5-hydroxymethyl uracil (5-hmU). 5-hmU is one of the most enigmatic oxidative modifications of DNA, mainly formed by the oxidation/hydroxylation of thymine or ROS reaction with 5-methylcytosine (Olinski et al., 2016). It is a common oxidative DNA lesion; however, specific repair activities mainly through hmU-DNA glycosylase remove this modified base from DNA to limit mutagenesis, cytostasis, and cytotoxicity (Cooke et al., 2003). Thus, oxidative DNA damage has been implicated in cancer and aging. Our data suggest that metformin can alter the DNA lesion repair mechanisms, explaining its association with reduced cancers (Olinski et al., 2016). Interestingly, we have previously reported a dysregulation in the level of 5-hmU in metformin-treated diabetic patients, highlighting the importance of this metabolite in the underlying mechanisms of metformin (Aleidi et al., 2021).

There is growing evidence that abnormalities in the microbiota composition may contribute to the development of non-communicable diseases, including diabetes and obesity (Lazar et al., 2019). The composition of the gut microbiota (diversity or the abundance of particular species) is defined by a combination of factors including gender, age, body mass index, host genetics and immunity, and therapeutics drugs (Maurice et al., 2013; Zhernakova et al., 2016). It has been reported that some actions of metformin may be mediated by altering the gut microbial diversity and enriching beneficial bacteria (Tong et al., 2018; Arneth et al., 2019). In the current work, the levels of two microbial metabolites, ethylphenyl sulfate, and propionic acid, changed similarly to the metformin pattern. Propionic acid is a short-chain fatty acid mainly produced by the fermentation of undigested food by the colonic microbiota. Propionic acid inhibits lipolysis and induces lipogenesis in adipose tissue, suppresses fatty acid production in the liver, inhibits food intake, increases the duration of satiety, and exerts immunosuppressive actions (Al-Lahham et al., 2010; Lazar et al., 2019). These beneficial effects are associated with improved insulin sensitivity and reduced body weight (Al-Lahham et al., 2010). Unlike propionic acid, the function of ethylphenyl sulfate in the host physiology and pathophysiology is not yet elucidated (Lazar et al., 2019). The association between propionic acid and metformin levels detected in the current study suggests that metformin might acutely boost the capability of the gut bacteria to produce certain types of short-chain fatty acids such as propionic acid, which intern can suppress the appetite and alter blood glucose levels in different ways.

Dysregulation in lipid metabolism and increased lipogenesis have been recognized as a hallmark of cancer and linked with PCOS disorder (Menendez and Lupu, 2007). Metformin-dependent metabolites acutely changed in an opposed manner to metformin patterns were mainly lipids and lipid-like molecules, including polyunsaturated fatty acids (PUFA), eicosanoids, and glycerophospholipid (Figure 4). Several studies have shown that enzymes involved in the glycerophospholipid pathway may be used as potential targets for antitumor therapy (Dolce et al., 2011). In highly proliferating cancer cells, *de novo* fatty acids synthesis continually provides glycerophospholipids essential for membrane production (Kuhajda et al., 1994). The fact that metformin significantly decreased the level of several glycerophospholipids herein suggests that one mechanism by which metformin inhibits cancer cell growth could be by altering key enzymes involved in glycerophospholipid synthesis. Moreover, in line with our data, the downregulation effect of metformin on glycerophospholipids has been reported in women with PCOS. The latter might contribute to the reported favorable effect of metformin on dyslipidemia in PCOS (Pradas et al., 2019).

Eicosanoids, including prostaglandins (PG), leukotrienes, and hydroxyeicosatetraenoic acids (HETEs), are generated from the metabolism of arachidonic acid by cyclooxygenase (COX), lipoxygenase (LOX), and cytochrome 450 pathways (Wang and Dubois, 2010). Eicosanoids and related bioactive lipid mediators derived from PUFA are complex and challenging lipid network signaling. Eicosanoids are much appreciated for their pleiotropic effects and implication in various pathological conditions, including inflammation and cancer (Wang and Dubois, 2010; Dennis and Norris, 2015). Many HETE metabolites are well-known pro-inflammatory eicosanoids that increase inflammatory cytokine expression inducing chronic inflammation associated with insulin resistance, metabolic syndrome, and T2DM (Chakrabarti et al., 2009; Guadarrama-Lopez et al., 2014). Additionally, pro-inflammatory eicosanoids, including PG and leukotrienes, can modulate tumor proliferation, apoptosis, migration, and invasion through multiple signaling pathways, and can remodel the tumor microenvironment with enhanced tumour angiogenesis (Wang and Dubois, 2010). In the current work, the levels of several metformin-dependent metabolites involved in the eicosanoid synthesis pathways, such as several HETEs and PGH involved in LOX and COX pathways, respectively, were decreased upon metformin administration and reserve to baseline level at 36-h post-dose. The presented findings indicate that the promising roles of metformin in improving insulin resistance, preventing cancer, and suppressing tumor progression might involve key enzymes in arachidonic acid metabolism particularly COX and LOX pathways. Our results are in line with (Hyun et al., 2013), who reported that metformin suppressed the protein level of COX-2 and may attenuate inflammatory responses. Moreover, recent *in vitro* and *in vivo* studies showed that a combination treatment of celecoxib (selective COX-2 inhibitor) and metformin inhibited the proliferation of Hepatocellular carcinoma to a greater extent than either treatment alone (Hu et al., 2020). However, there is little known about metformin’s effects on lipid and arachidonic acid metabolisms, and more in-depth studies to investigate its impact on these pathways remain urgent.

Several acylcarnitines showed an increase in their level that was independent of metformin level, Figure 5C. The level of these metabolites was initially decreased upon metformin administration and then linearly increased to return to normal level at 36 h post metformin administration. This suggests that the change in acylcarnitines level might be context-dependent and affected by factors other than metformin level, such as the pathological state of the subject.

Acylcarnitines are derived from l-carnitine esterification by carnitine acyltransferases during the β-oxidation of fatty acids in the mitochondria (Houten and Wanders, 2010). Several studies have demonstrated the importance of l-carnitine and acylcarnitines in fatty acid oxidation and the modulation of intracellular coenzyme A (CoA) homeostasis, maintaining normal mitochondrial functions (Reuter and Evans, 2012). These functions crucially influence physiological processes, including energy homeostasis, regulation of insulin secretion, and insulin sensitivity (Mai et al., 2013). Disturbances in the endogenous carnitine pool (l-carnitine and short, medium, and long-chain acylcarnitines) serve as a diagnostic marker for the equilibrium between acyl-CoA and acylcarnitine species. They can reflect mitochondrial and metabolic dysfunction (Reuter and Evans, 2012). Increased acylcarnitine levels have been proposed as markers of insulin resistance, T2DM and cardiovascular diseases (Koves et al., 2008). Our findings indicate that metformin might initially affect fatty acid β-oxidation by altering carnitine palmitoyltransferase activity and decreasing acylcarnitines’ levels.

Coumarinic acid is another metformin-independent metabolite that increased upon metformin administration and then decreased to a normal level at 36 h post-dose. Coumarinic acid is a hydroxycinnamic acid derivative and one of the three isomers of coumaric acid: o-coumaric acid, m-coumaric acid, and p-coumaric acid. This class of phenolic compounds has been reported to possess potent antioxidant and anti-inflammatory properties both *in vitro* and *in vivo* by decreasing the expression of inflammatory mediator TNF-α and circulating immune complexes (Pragasam et al., 2013; Alam et al., 2016). Additionally, coumaric acids have been reported to exhibit marked antidiabetic action (Omar et al., 2016; Abdel-Moneim et al., 2018). o-coumaric acid or coumarinic acid was found to restore glycemic control and insulin sensitivity in rats fed high fats/high sucrose diet and suffered from hyperglycemia and insulin resistance (Omar et al., 2016).

Numerous favorable effects have been reported for metformin. Several lines of evidence implicate that metformin possesses hepatoprotective effect from injuries induced by hepatotoxic substances or liver conditions (Iranshahy et al., 2019). Alkaline phosphatase (ALP) is a well-known biomarker for liver disease, and its elevated blood level has been linked with liver injury (Saravi et al., 2016). In our work, metformin was associated with a significant decrease in the level of ALP, while no significant effect was detected on aminotransferase levels GOT and GPT (Table 1). This finding can be linked with the previously reported hepatoprotective effect of metformin, where metformin was found to normalize or decrease the levels of ALP enzyme (Saravi et al., 2016). Noteworthy, metabolomics data identified one subject with different metabolic profiles, even before metformin administration, that had a significant increase in the liver enzymes after metformin intake. This finding points to the fact that individuals may vary in response to this therapy according to their metabolic pattern and highlights the important role of metabolomics in personalized medicine.

Of note, the identified metabolites linked to metformin intake herein were dysregulated after a single dose of metformin. Therefore, a follow-up study in the future under chronic conditions remains essential to evaluate and validate their link to metformin pharmacodynamics, and their role as potential biomarkers to monitor the pharmacological effect of metformin.

## Conclusion

In the present study, MS-based untargeted metabolic profiling was applied for the first time to uncover the biochemical changes induced by metformin at different time points in healthy subjects.

Our findings revealed that BCAA pathway was the most significantly altered pathway by metformin. Additionally, specific metabolites that showed metformin-dependent changes in their levels were identified, including 5-hmU, propionic acid, and several eicosanoids. The altered metformin-dependent metabolites pointed to fundamental biochemical processes by which metformin can exert its multiple beneficial effects, including lipid network signaling, inflammation, energy homeostasis, DNA lesion repair mechanisms, and gut microbiota functions. Thus, the distinctive metabolic pattern linked to metformin intake, particularly metformin-dependent metabolites, can be used as potential biomarkers or metabolic signature to predict the potential effect and mechanism of actions of new chemical entities during drug development.

Further studies with larger sample sizes and under chronic conditions are necessary to evaluate the link between the dysregulated metabolites and metformin pharmacodynamics and investigate their role as potential biomarkers to monitor the pharmacological effect of metformin. Once validated, metformin-dependent endogenous metabolites might be utilized in conjunction with other existing biomarkers to monitor metformin's efficacy for personalized medicine, especially since metformin is not metabolized inside the body and is excreted unchanged urine.

Future research is warranted to investigate the effect of metformin on BCAA pathway and, most importantly, to identify the endogenous lipid pattern induced by metformin. Refined lipidomics methodologies can be applied to provide a deeper understanding of the role of metformin in arachidonic acids metabolism and eicosanoids signaling and identify new potential targets for inflammatory and metabolic conditions.

## Data Availability

The raw data supporting the conclusions of this article will be made available by the authors, without undue reservation, to any qualified researcher.
